# Network Substrates of Centromedian Nucleus Deep Brain Stimulation in Generalized Pharmacoresistant Epilepsy

**DOI:** 10.1007/s13311-021-01057-y

**Published:** 2021-04-26

**Authors:** Cristina V. Torres Diaz, Gabriel González-Escamilla, Dumitru Ciolac, Marta Navas García, Paloma Pulido Rivas, Rafael G. Sola, Antonio Barbosa, Jesús Pastor, Lorena Vega-Zelaya, Sergiu Groppa

**Affiliations:** 1 Department of Neurosurgery, University Hospital La Princesa, Madrid, Spain; 2 Movement Disorders and Neurostimulation, Department of Neurology, Focus Program Translational Neuroscience (FTN), University Medical Center of the Johannes Gutenberg University Mainz, Rhine Main Neuroscience Network (rmn2), Mainz, Germany; 3 Laboratory of Neurobiology and Medical Genetics, Nicolae Testemitanu, State University of Medicine and Pharmacy, Chisinau, Republic of Moldova; 4 Department of Neurology, Institute of Emergency Medicine, Chisinau, Republic of Moldova; 5 Department of Neuroradiology, University Hospital La Princesa, Madrid, Spain; 6 Department of Clinical, Neurophysiology University Hospital La Princesa, Madrid, Spain

**Keywords:** Deep brain stimulation, Brain networks, Centromedian nucleus, Neuromodulation, Generalized epilepsy

## Abstract

Deep brain stimulation (DBS), specifically thalamic DBS, has achieved promising results to reduce seizure severity and frequency in pharmacoresistant epilepsies, thereby establishing it for clinical use. The mechanisms of action are, however, still unknown. We evidenced the brain networks directly modulated by centromedian (CM) nucleus-DBS and responsible for clinical outcomes in a cohort of patients uniquely diagnosed with generalized pharmacoresistant epilepsy. Preoperative imaging and long-term (2–11 years) clinical data from ten generalized pharmacoresistant epilepsy patients (mean age at surgery = 30.8 ± 5.9 years, 4 female) were evaluated. Volume of tissue activated (VTA) was included as seeds to reconstruct the targeted network to thalamic DBS from diffusion and functional imaging data. CM-DBS clinical outcome improvement (> 50%) appeared in 80% of patients and was tightly related to VTAs interconnected with a reticular system network encompassing sensorimotor and supplementary motor cortices, together with cerebellum/brainstem. Despite methodological differences, both structural and functional connectomes revealed the same targeted network. Our results demonstrate that CM-DBS outcome in generalized pharmacoresistant epilepsy is highly dependent on the individual connectivity profile, involving the cerebello-thalamo-cortical circuits. The proposed framework could be implemented in future studies to refine stereotactic implantation or the parameters for individualized neuromodulation.

**Supplementary Information:**

The online version contains supplementary material available at 10.1007/s13311-021-01057-y.

## Introduction

Epilepsy is a very common chronic neurological disorder characterized by spontaneous recurrent seizures, presenting a high prevalence and leading to an enormous psychosocial burden for patients, families, caregivers, and health systems [1]. Approximately 30% of epilepsy patients will not have adequate seizure control with pharmacotherapy alone [1] and long periods of incomplete seizure control have considerable consequences leading to disease worsening, cognitive and mental symptoms, and a massive decline in quality of life.

Recent work brought first important hints for the mechanisms underlying generalized pharmacoresistant epilepsy, showing widespread reduced structural integrity, within the frontal, sensorimotor, and parietal cortices, as well as the anterior cingulate [2, 3], which accelerates in patients with poorly controlled seizures [2]. EEG-fMRI studies have shown that during generalized seizures, a characteristic pattern of subcortical (medio-dorsal thalamic and striatum) activation and cortical deactivation occurs [4]. Particularly, the activation of the cortico-reticular (centromedian [CM] nucleus of the thalamus and parafascicular [Pf]) nuclei of the thalamus precede the activation of the anterior nucleus, suggesting that the CM-Pf complex as driving the generation, or early propagation of generalized seizures, while anterior nucleus activity supports its maintenance [4]. For other structures, such as the cerebellum and brainstem, despite the available evidence, their particular role is less clear. A potential antiseizure modulatory effect of the cerebellum can be postulated as reduced cerebellar functional connectivity is related to pharmacoresistance [5]. These findings serve to postulate a network state associated with the pathophysiology of generalized pharmacoresistant epilepsy. However, its specific attribution for the therapeutic interventions in generalized pharmacoresistant epilepsy remains to be elucidated.

Deep brain stimulation (DBS) of the CM has been recently introduced as a safe and promising therapy in patients with pharmacoresistant epilepsy [6]. The efficacy of CM-DBS may, however, depend on the epilepsy syndrome, i.e., possibly being more effective in patients with generalized than focal epilepsy [6]. First insights into CM-DBS efficiency and way of action have been obtained from studies in patients with mixed seizure types [7] or Lennox-Gastaut syndrome [8]. Thus, a conceptual framework of CM-DBS in pharmacoresistant epilepsies and mainly in generalized forms is still lacking. Moreover, stratification algorithms to identify optimal candidates for CM-DBS are still pending. We postulate that the evaluation of the connectivity profiles of CM-DBS will unmask a robust neuroanatomical substrate common for all patients. Such substrate can be identified from associations between individual seizure reductions and the connectivity profile of the targeted network. Increasing evidence shows that stimulation of white matter tracts is, at least in part, responsible for the therapeutic effects of DBS in network disorders [9, 10]. Connectivity, thus, can be used for the target definition in stereotactic implantation or identification of surgery candidate patients. For this reason, we use structural and functional connectivity as a main tool for delineating the networks directly associated with CM-DBS clinical outcomes in patients with generalized pharmacoresistant epilepsy. This strategy will further not only reduce the heterogeneity across patients and studies [11, 12] but also elucidate the physiological and mechanistic substrates of CM-DBS. If available, this information could be used to yield optimal clinical efficacy for this therapy and translation into clinical practice through adjustment of stimulation parameters.

## Material and Methods

### Patients

We conducted a retrospective study of 10 patients with generalized pharmacoresistant epilepsy (mean age at surgery = 30.8 ± 5.9 years, 4 female), defined according to the International League Against Epilepsy (ILAE) guidelines [13], who have undergone CM-DBS at our institution between 2008 and 2019. Day-to-day functioning was evaluated through the Global Assessment of Functioning scale (GAF) [9], and quality of life (QoL) was evaluated through the Diagnostic and Statistical Manual of Mental Disorders (DSM-IV) [9, 14].

A multidisciplinary team, consisting of neurosurgeons, epileptologists, psychiatrists, neurophysiologists, radiologists, and neuropsychologists, determined the indications for the DBS. Table 1 depicts the specific inclusion and exclusion criteria for the study. In eight patients, before the DBS, vagal stimulation implantation was performed with short-lasting beneficial response. Each patient’s family completed the diary of seizures. In all cases, there was a co-existing developmental delay. Prior to surgery, patients were evaluated through medical history, physical examination, magnetic resonance imaging (MRI), ^99^mTc-HmPAO single-photon emission tomography (SPECT), 19 electrodes scalp electroencephalography (EEG) (Cadwell®, Kennewick, WA, USA), and video-EEG (VEEG, XLTEK®, Oakville, ON, Canada). EEG electrodes were placed according to the 10–20 system, including supplementary electrodes at bilateral basal temporal lobes. VEEG was used to determine the seizure type, frequency, and electro-clinical features.

**Table 1:** Inclusion and exclusion criteria

Inclusion criteria	Exclusion criteria
• Age > 18 years. • A clear diagnosis of epilepsy (confirmed by surface or intracranial VEEG). • Patients were not candidates for resective surgery. • Seizure frequency greater than 10/month. • Stable doses of antiepileptic drugs in the last 6 months. • The family was able to fill out the diary of seizures. • No structural abnormalities in MRI that could impact centromedian nucleus targeting or its connections.	• Concomitant neurological or psychiatric disorders (although epileptic encephalopathy is not excluded). • A history of poor adherence to treatment. • Temporal lobe epilepsy.

### Surgical Procedure

DBS implantation into the CM was performed under general anesthesia with propofol and isoflurane using an MRI-guided stereotactic protocol, previously described elsewhere [15]. In brief, a stereotactic frame (Leksell Frame G, Elekta, Stockholm, Sweden) was placed and used to determine the target coordinates (*X* = 9, *Y* = − 9, *Z* = 0). After stereotactic frame positioning, correct targeting was evaluated according to the presence of a specific thalamic response to somato-sensory evoked potentials (delta waves) at the level of the cerebral cortex, induced by electrical stimulation at 6 Hz (monophasic pulse width 100 µs, amplitudes between 1 and 3 mA). Cortical responses were assessed by intraoperative microelectrode recordings (MER) using five microelectrodes implanted through bilateral frontal burr holes in a transparenchymal extraventricular trajectory and scalp EEG [16]. Following MER target verification, each patient was implanted with two quadripolar DBS electrodes (model: Medtronic 3389, Medtronic, Minneapolis, MN, USA, or Abbott 6149, St. Jude Medical Inc., Saint Paul, MN, USA) connected to pulse generators (Kinetra, Medtronic, Inc., Minneapolis, MN, U.S.A., and Libra PC, Abbott) placed in the subclavicular area. Final electrode positioning was revised with intraoperative radioscopy and with generation of delta waves on EEG at macrostimulation. No modifications in medical therapy were allowed during the first 12 months after CM-DBS. Postoperative whole-brain MRI was performed in all patients to verify the electrodes’ position and rule out surgery complications.

### CM-DBS Configuration

After CM-DBS implantation, patients were subsequently monitored by VEEG during 3–5 days. Optimal stimulation parameters and DBS active contacts were selected according to recorded cortical delta waves (6 Hz) generation [17]. CM-DBS was activated at 60 Hz and 90 μs, and up to 5 V, depending on the initially recorded responses. DBS was activated 3 months after DBS implantation in all patients; however, 60% of patients remained uninformed about this until the 6-month visit. Patients were clinically evaluated at three months before DBS activation, to inform about the confusion factor of the electrode insertion effect (“microlesion” or “honeymoon” effects of implantation) on the 6-month visit onwards. Between DBS activation and the 6-month visit, stimulators were individually adapted according to EEG improvement. Postoperative seizure frequency was assessed by seizure diaries and by VEEG during the first postoperative week and every 6 months over 2 years. The average follow-up time after CM-DBS was 92.4 months (42–129 months).

### MRI Acquisition

Whole-brain imaging data were acquired on a 1.5-T MRI scanner (General Electric Healthcare, Waukesha, WI). Pre-operative diffusion data were acquired using a single-shot echo-planar imaging pulse sequence with following parameters: repetition time (TR) = 11 s; field of view (FoV) = 280 × 280 mm; matrix size = 128 × 128; slice thickness = 3 mm; voxel size 1 × 1 mm; 25 gradient directions and a *b*-value of 1000 s/mm^2^, and an additional volume without diffusion weighting (*b* = 0 s/mm^2^). A T2-weighted sequence was acquired using a 3D magnetization-prepared cube fast spin gradient echo (FoV = 25.6 mm, slice thickness = 1 mm, TR = 2500, echo train length = 100, bandwidth = 62.5 and matrix size = 256 × 256). Pre- and post-operative T1-weighted images were acquired using a 3D magnetization-prepared rapid gradient-echo (MPRAGE) sequence with the following parameters: voxel size of 1 × 1; slice thickness = 1 mm, FoV = 25.6 mm; matrix size = 256 × 256; TR = 8300; echo train length = 3100; bandwidth = 31.25.

### DBS Electrode Reconstruction and Localization

Image processing and electrode localization were carried out by using the Lead-DBS toolbox (v.2.3; https://www.lead-dbs.org/) with default parameters [18]. Briefly, preoperative and postoperative MRI scans were co-registered using a linear transform in SPM12 (http://www.fil.ion.ucl.ac.uk/spm/software/). Pre- and post-operative images were then normalized into MNI space (2009b non-linear asymmetric) using a fast diffeomorphic image registration algorithm (DARTEL) as implemented in SPM12 [19]. Brain shifts in postoperative acquisitions were corrected by applying the “subcortical refine” setting as implemented in the Lead-DBS [18]. Finally, DBS electrodes were manually localized based on the post-operative acquisitions by using the “display” tool in SPM12. All steps were visually inspected to ensure data quality. To graphically illustrate the electrode locations, two-dimensional slices were plotted using the 7-T 100-μm *ex vivo* human brain MRI [20] template as a background image and the thalamic nuclei boundaries as delineated in the THOMAS atlas [21] as reference.

### Estimation of VTA

Stimulation parameters, i.e., active contacts and amplitudes, of each individual patient were applied to calculate VTAs, representing a rough approximation of the surrounding tissue modulated by DBS, using a finite element method (FEM) approach [18]. Anisotropic conductivity values for gray (*σ* = 0.33S/m) and white matter (*σ* = 0.14S/m) were chosen. The electric field threshold was set to *e* = 0.2 V/mm, which approximates previous VTA radius estimates [18].

### Diffusion Imaging Pre-processing and Tractography

Diffusion MRI data underwent correction of eddy current distortions and subject movement, followed by registration to the corresponding T1 image using the normalized mutual information algorithm implemented in SPM12. Then, deterministic tractography was performed using the generalized Q-sampling imaging method from the DSI studio (http://dsi-studio.labsolver.org) using the default parameter sets implemented in the Lead-Connectome (www.lead-connectome.org). The resulting whole-brain set of 200.000 fiber tracts in the patient space were transformed into MNI (ICBM 2009b Nonlinear Asymmetric) space and merged into one whole-brain connectome [18].

### Resting-State Functional Imaging

Normative resting-state fMRI was obtained from 1000 healthy subjects using a 3-T Tim Trio MRI scanner (Siemens Healthcare, Erlangen, Germany) with a 12-channel receive only coil, as part of the publicly available Brain Genomics Superstruct Project (GSP) [22]. fMRI data were acquired at 3-mm isotropic resolution with TR = 3000 ms and 124 frames. fMRI data pre-processing included: (1) removal of the first five frames, (2) motion correction using rigid body translation and rotation, (3) slice timing correction, (4) alignment with structural image, (5) normalization to MNI space using the deformation matrices obtained during MRI preprocessing using the CAT12 toolbox (Structural Brain Mapping group, Jena University Hospital, Jena, Germany), (6) smoothing by a 6 mm full-width half-maximum (FWHM) kernel, (7) nuisance covariate regression (including six motion correction parameters, and averaged WM and CSF signals), and (8) band-pass filtering (between 0.01 and 0.08 Hz). WM and CSF masks were obtained from segmentation of the anatomical T1 image, followed by binarization of the probabilistic tissue maps at a threshold of 0.9 and 0.7, respectively. All preprocessing steps were carried out following recommended guidelines [23] in SPM12.

### Connectivity Analysis

VTAs were used as seeds to estimate diffusion- and fMRI-based connectivity to other brain areas. For diffusion imaging, only fibers that traversed through the VTA and terminated in distinct brain regions defined according to the Harvard–Oxford atlas [24] were selected. Next, we used a method referred to as “discriminative fibertract analysis” [25] to select the fibers that are strongly discriminative for better clinical outcomes across patients [26]. Briefly, for each fiber connecting the VTAs with the rest of the brain, the algorithm searches whether the fiber passes close to an active contact of patients with optimal seizure improvement and is far from contacts of patients with poor improvement. This search results in a “statistical” score assigned to the fiber (see statistical analyses for details on scores). High scores mean that a particular fiber has a strong discriminative value for the clinical outcome.

For functional connectivity, the time series sampled from VTA voxels were spatially correlated (Pearson’s product-moment correlation) with the time series from every other voxel in the brain for each of the 1000 normative images. Then the individual correlation maps were *z*-transformed using Fisher's transformation and used to compute a whole-brain connectivity t-map.

### Statistical Analysis

Statistical comparisons between preoperative and postoperative clinical variables were conducted under the general linear model (GLM) by firstly fitting a repeated measures ANOVAs (rm-ANOVA) with 95% confidence (*p* < 0.05), followed by pairwise post hoc comparisons by means of Tukey–Kramer significance difference criterion. Associations between volume intersections (between each patient’s bilateral contact coordinates/VTAs and the bilateral CM) and clinical outcomes were evaluated by setting linear regression analysis (two-sided) under the general linear model. Here, *r* coefficients are presented as indicators of the effect sizes. Streamline scoring during the fibertract discriminative analysis was effectuated by conducting mass-univariate two-sided tests, comparing improvement values of connected VTAs against those of unconnected VTAs. Thus, in this step, each fiber receives a discriminative value in form of a *t*-score that can be positive (indicating fibers predominantly connected to VTAs that are associated with better treatment response) or negative (indicating the opposite). Based on this *t*-score, only the top 10% of all discriminative fibers were kept [26]. All statistical analyses were conducted in Matlab (R2017b, The MathWorks®).

## Results

### Patient Evaluation

Table 2 summarizes baseline characteristics of the included patients with generalized pharmacoresistant epilepsy. All patients had generalized epileptiform discharges on EEG recordings. Among other seizure types, the majority of patients presented generalized tonic–clonic seizures. No patient had post-surgical complications, nor paresthesia.

**Table 2:** Patient demographic and clinical baseline characteristics

Age at surgery	Daily seizure frequency	Dominant seizure type (VEEG)	Further seizure types	SB (baseline)	SB (DBS-OFF)	SB (DBS-ON)	QoL-DSM-IV	Last follow-up (months)
30 yrs	2–30	Myoclonic	Abscence, atonic	15	10	2	20	88
35 yrs	4	TCG	n/a	265	45	15	40	124
21 yrs	1	Tonic	Abscence	180	80	32	30	71
39 yrs	1	TCG	n/a	7200	7200	7200	30	123
39 yrs	1–20	Spasms	Myoclonic, tonic	1000	2000	40	70	6
30 yrs	10–20	Atonic	Abscence	75	54	33	50	129
27 yrs	4–10 (clustered, weekly mean)	TCG	n/a	60	1.5	30	20	52
25 yrs	1	TCG	n/a	90	130	12	40	124
28 yrs	2–21	TCG	n/a	584	584	340	50	42
34 yrs	1–4	Atonic	Abscence, myclonic	240	120	60	40	79

*VEEG *= video-electroencephalogram; *TCG*  = tonic–clonic generalized seizures; *SB*  = seizure burden (sec/day); *QoL*  = quality of life assessed through by the Diagnostic and Statistical Manual of Mental Disorders Z(DSM-IV); *n/a *= not available

**Fig. 1:**
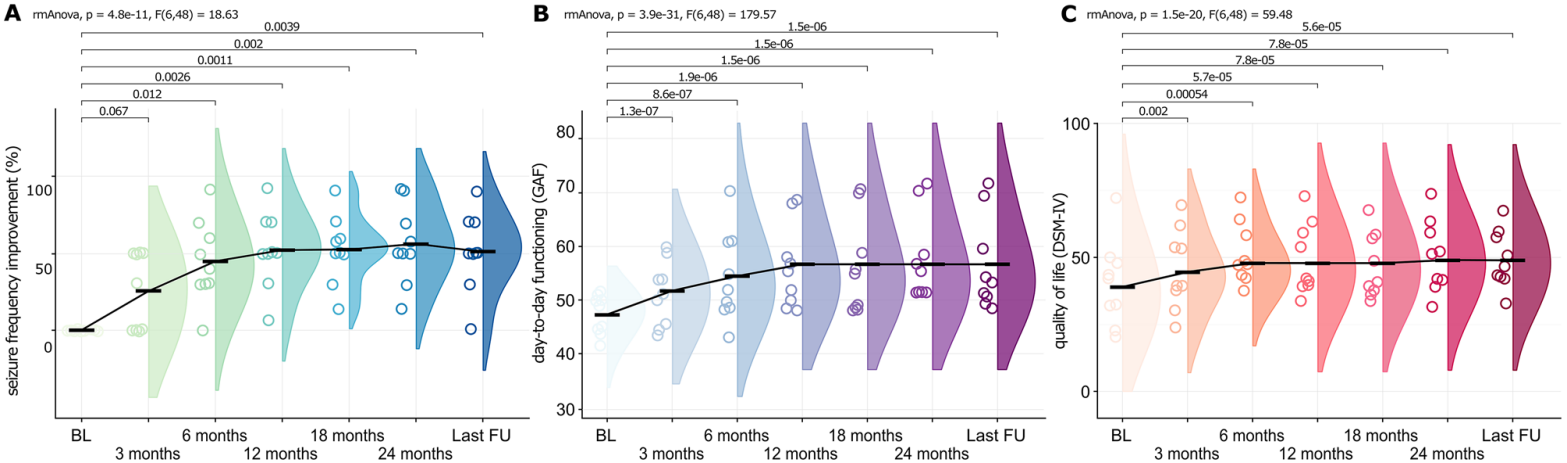
Long-term clinical improvement after CM-DBS. (**a**) Seizure frequency improvement presented as percentage in comparison to baseline (BL). (**b**) Day-to-day functioning measured through the Global Assessment of Functioning (GAF). (**c**) Quality of life measured by the Diagnostic and Statistical Manual of Mental Disorders (DSM-IV). All graphs depict an overall improvement for the patients after CM-DBS. The minimum follow-up (FU) for all patients was 24 months, whereas the last FU ranged from 42 to 129 months

After CM-DBS eight of the ten patients (80%) presented a decrease in seizure frequency of at least 50% (Fig. 1a). Compared to baseline and to the pre-DBS activation period (three months), seizure frequency significantly improved in time (mean % improvement, 3 months = 25%, 6 months = 45%, 12 months = 52%, 18 months = 52%, 24 months = 56%, last follow-up = 51%). rmANOVA across all time points (*p* = 4.8e−11, *F*(6,48) = 18.63) (Fig. 1a). The last follow-up varied in time among the subjects (> 3 and up to 10.8 years). Although, the clinical outcomes were comparable between the 24 months follow-up and the last follow-up (all *p* > 0.05), in order to avoid confounding effects of variable therapy duration at last follow-up, improvement at the 24-month follow-up was used for the interpretation of long-term time effects of CM-DBS. Significant clinical improvement at 24 months was evidenced compared to baseline (post hoc *p* = 0.002) and 3-month (post hoc *p* = 0.048) data. Post hoc analyses showed no further differences across time points. These findings suggest that seizure improvement in all clinical assessments after 6 months was achieved in comparison to the first three months after the DBS surgery, thus excluding any electrode insertion effect (“microlesion” or “honeymoon” effects of implantation) on further follow-up assessments.

Patients’ evaluation further evidenced similar effects after CM-DBS in the day-to-day functioning (Fig. 1b) and quality of life (Fig. 1c), evaluated through the GAF and DSM-IV, respectively. Significant improvement across all follow-ups was observed for GAF (rmANOVA *p* = 3.9e−31, *F*(6,48) = 179.57), and for QoL (*p* = 1.5e−20, *F*(6,48) = 59.48). Post hoc evaluation between 24-month follow-up with the baseline was significant for both GAF (*p* = 1.54e−06) and QoL (*p* = 7.8e−5). Post hoc evaluation between the 24-month follow-up with the 3-month follow-up was also significant (GAF *p* = 0.022; QoL *p* = 0.042). Accompanying the findings on DSM-IV, improvement in quality of life of patients was reflected as (i) less interference in daily activities due to seizures; (ii) fewer hospital admissions due to status epilepticus, aspiration pneumonia, and hyperthermia; (iii) less time in post-critical period; and (iv) cognitive improvement in three patients, so they were more interactive with their families and independent to perform their daily activities. Indeed, one of the patients was able to go by bus to school, whereas that was considered impossible in the preoperative period, thus supporting the observed clinical improvement.

### Electrode Localization and Clinical Outcome

**Fig. 2:**
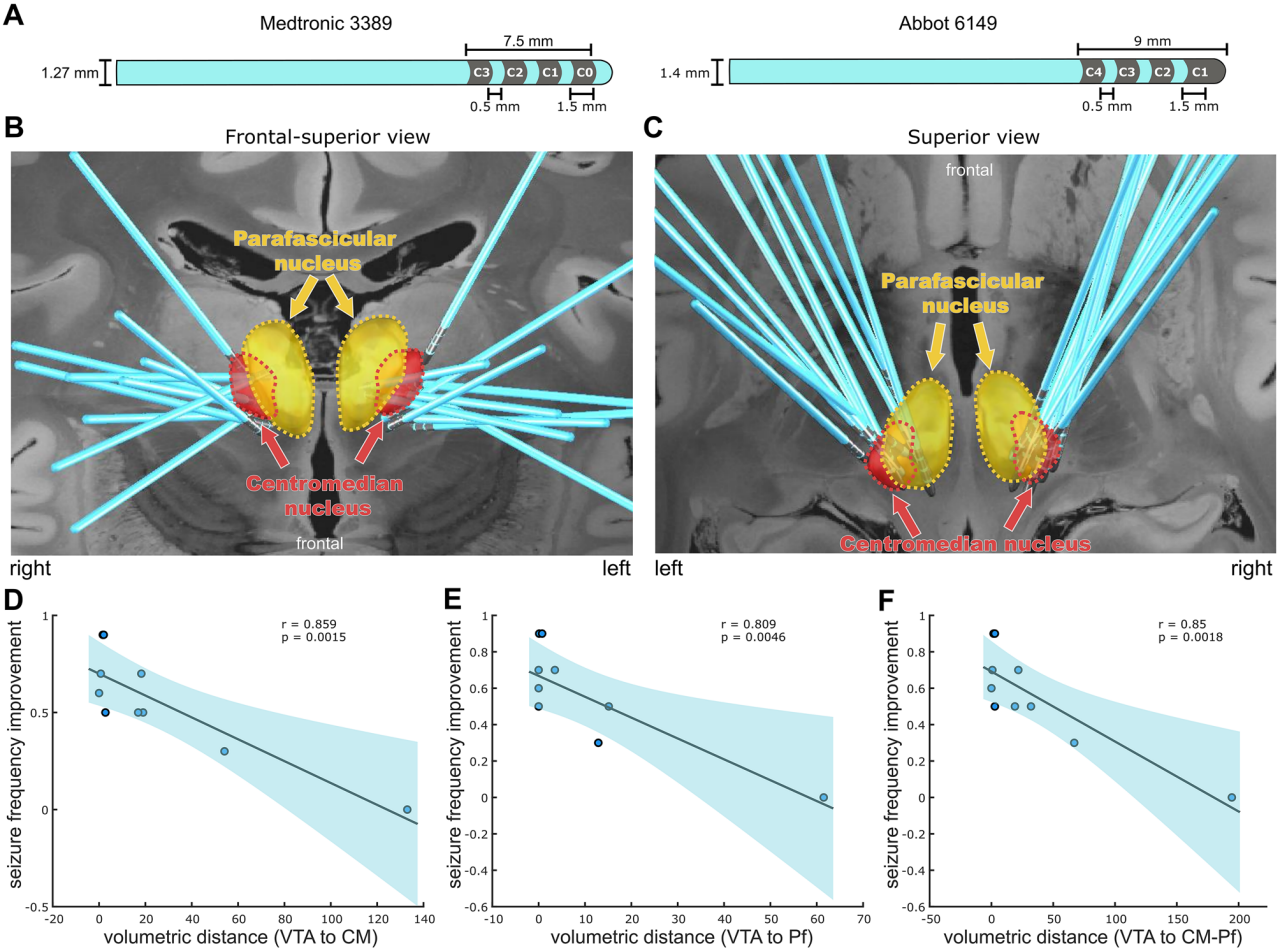
CM-DBS localization overview. (**a**) Schematic representation of DBS electrodes. (**b**) and (**c**) Frontal and superior 3D view representations of the DBS contact locations. The THOMAS atlas [21] was used as reference for delimitation of the thalamic centromedian (CM, red) and parafascicular (Pf, yellow) nuclei. A 7T 100-μm T1 MRI scan of an *ex vivo* human brain [20] is used as the background image for 2D slices. (**d**) Association between the volumes of tissue activated (VTA) and the CM location with seizure frequency improvement (%). (**e**) Association between VTA and Pf location with seizure frequency improvement (%). (**f**) Association between VTA and combined CM/Pf location with seizure frequency improvement (%)

Schematic depiction of implanted DBS electrodes, including lead width, contact length, and intercontact distance is presented in Fig. 2a. In brief, each lead has four stimulation contacts (C0, C1, C2, and C3) spaced 0.5 mm apart. Electrode localization confirmed accurate placement of the electrode leads within the target region in the thalamus (CM and Pf) in all patients (Fig. 2 b and c). However, there was some observable heterogeneity across individuals, mostly in the left hemisphere. Such heterogeneity is not surprising as it clearly follows the overall variability in the clinical DBS outcomes. Particularly, the optimal DBS outcome was associated with shorter distances between VTA and CM (*r* = 0.859, *p* = 0.0015; Fig. 2d) and to a lesser extent between VTA to Pf (*r* = 0.809, *p* = 0.0046; Fig. 2e). The distances to combined CM-Pf also correlated to seizure improvement (*r* = 0.85, *p* = 0.0018; Fig. 2f).

### Connectivity Analysis and Clinical Outcomes

**Fig. 3:**
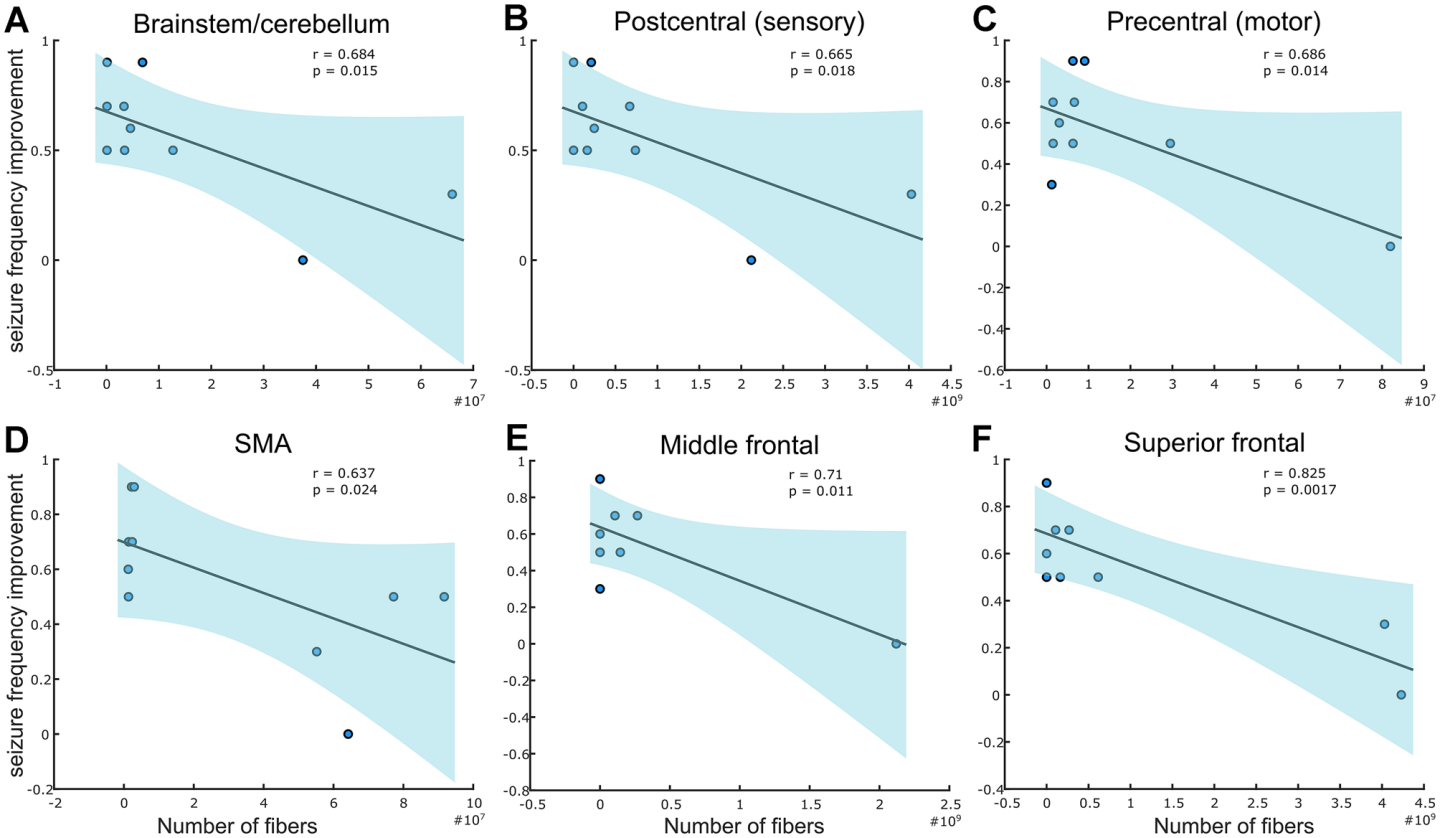
Association between CM-DBS and clinical outcome. Regression plots for the association between the seizure frequency improvement and the CM-DBS-modulated number of fibers with the (**a**) brainstem, (**b**) postcentral (sensory) cortex, (**c**) precentral (motor) cortex, (**d**) supplementary motor area (SMA), (**e**) middle frontal gyrus, and (**f**) superior frontal cortex. All associations were conducted using independent general lineal models. Blue shaded areas represent 95% confidence intervals

**Fig. 4:**
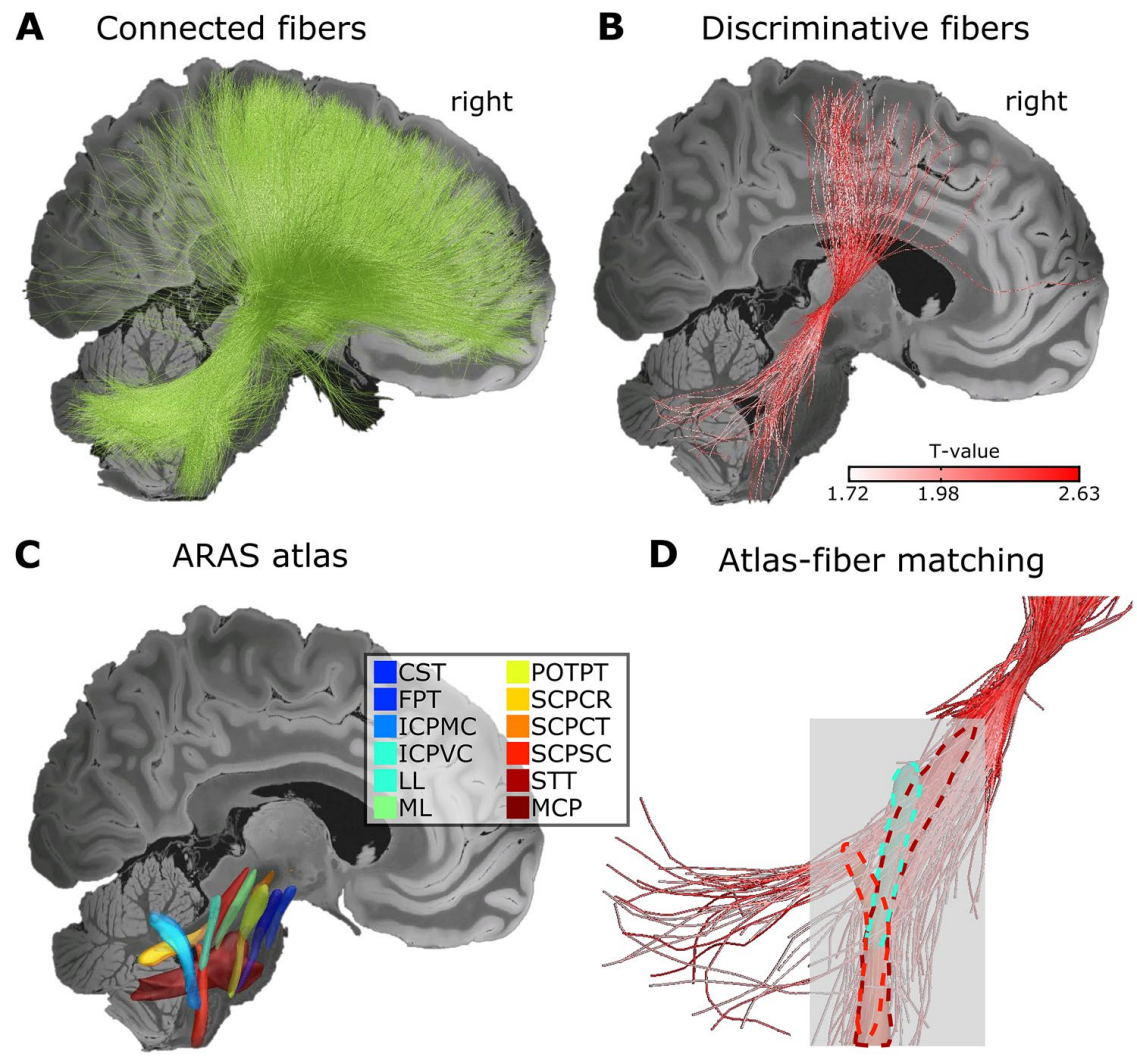
Delineation of CM-DBS structural network connectivity. (**a**) All fibers connected to the volumes of tissue activated (VTAs) across patients. (**b**) Discriminative fibers associated with clinical seizure improvement. The top 10% predictive fibers are displayed. Fibers in white to red scale represent the *t*-values for the positive association between selected fibers and seizure frequency improvement. Fibers with the strongest discriminative value cross from the VTAs in the centromedian nucleus (CM) to the brainstem and cerebellum. On the cortical side, these fibers project to the sensorimotor and supplementary motor cortices. Among cerebellar/brainstem fibers, projections occur in a similar fashion as the ascending reticular activating system (ARAS) as depicted in the Brainstem Connectome Atlas [27] (**c**), with the highest overlap with spinothalamic (STT), followed by the lateral lemniscus (LL) and superior cerebellar peduncle spinocerebellar (SCPSC) tracts (**d**). A 7 T 100-μm T1 MRI scan of an *ex vivo* human brain [20] is used as background image for 2D slices. CST corticospinal tract, FPT fronto-pontine tract, ICPMC inferior cerebellar peduncle medulla oblongata cerebellar, ICPPVC inferior cerebellar peduncle vestibulocerebellar, ML medial lemniscus, POTPT parieto-occipito-temporo-pontine tract, SCPCR superior cerebellar peduncle cerebellorubral, SCPCT superior cerebellar peduncle cerebellothalamic, MCP middle cerebellar peduncle

Analysis of the structural connectome revealed a high number of fibers connecting the VTA to the brainstem/cerebellum (mean count across subjects ± SD = 1.35e−7 ± 2.2e−7), postcentral cortex (8.29e−8 ± 1.3e−9), precentral cortex (1.47e−7 ± 2.5e−7), supplementary motor area (SMA; 3e−7 ± 3.7e−7), middle frontal gyrus (2.64e−8 ± 6.6e−8), and superior frontal cortex (9.41e−8 ± 1.7e−8). CM-DBS-related seizure frequency improvement was associated with the number of connecting fibers to the brainstem/cerebellum (*r* = 0.684, *p* = 0.015), postcentral cortex (*r* = 0.665, *p* = 0.018), precentral cortex (*r* = 0.686, *p* = 0.0.014), supplementary motor area (*r* = 0. 637, *p* = 0.024), middle frontal gyrus (*r* = 0.0.71, *p* = 0.011), and superior frontal cortex (*r* = 0.825, *p* = 0.0017) (Fig. 3). Of notice, repetition of the analysis without the patient not responding to CM-DBS did not change the results (see Supplementary file 11 Fig. 1). These results indicate that a particular proportion of these fiber projections and not all connected fibers (Fig. 4a) are responsible for CM-DBS clinical outcome. In order to test this hypothesis, the discriminative fiber analysis was conducted, evidencing that among connected fibers those projecting from the VTA to the brainstem and traversing to the cerebellum, together with the fibers connecting to the sensorimotor and supplementary motor cortices are tightly associated with optimal CM-DBS outcome (Fig. 4b). These discriminative fiber tracts overlap with the ascending reticular activating system (ARAS [27]; Fig. 4c), particularly corresponding greatly to the spinothalamic tract (STT), and as well to the superior cerebellar peduncle anterior spinocerebellar tract (SCPSC) and the lateral lemniscus (LL) (Fig. 4d).

**Fig. 5:**
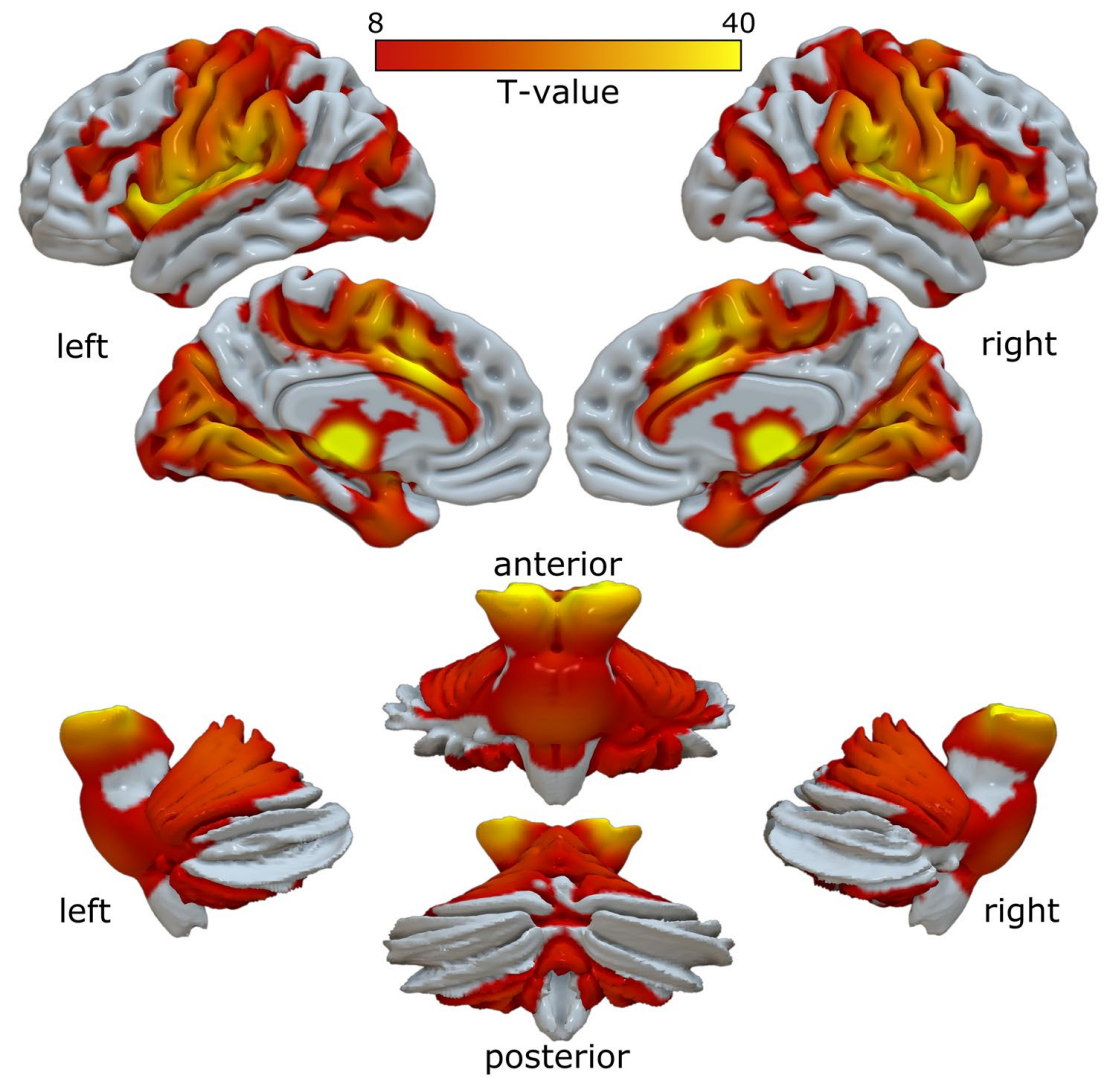
The CM-DBS-targeted functional network connectivity. Functional connectivity CM-DBS showed a very symmetric pattern across cerebral hemispheres that closely reproduced the structural connectivity pattern. The red to yellow color bar depicts the intensity of the connectivity from VTA to the rest of the brain. Specifically, the strongest connectivity appeared in the surrounding thalamic nuclei, followed by the brainstem and spreading to the cerebellum. Cortically, strong connectivity was detected in the anterior cingulate cortex, extending to the supplementary motor areas, the precentral and postcentral gyri, middle frontal cortex, and insula. Finally, connectivity was also depicted in the medial temporal and occipital cortices

For functional connectivity, positive connectivity was found with the cerebellum and brainstem. Cortically, connectivity was detectable with the sensorimotor cortex, SMA, middle frontal cortex, medial temporal cortex, and anterior cingulate. Subcortically, positive connectivity was found with the thalamus, extending to the striatum (globus pallidus, putamen, and caudate) and subthalamic nucleus. No areas of negative connectivity were found (Fig. 5). Thus, the patterns of functional connectivity are markedly similar to the structural connectivity results.

Additionally, associations between CM-DBS stimulation intensity and seizure frequency improvement (*r* = 0.78, *p* = 0.012) and between stimulation intensity and VTAs (*r* = 0.85, *p* = 0.0037) were further attested.

## Discussion

Our results show that the connectivity patterns of the CM-DBS-modulated fiber tracts in generalized pharmacoresistant epilepsy are responsible for the reduction of seizure frequency and, hence, improved clinical outcomes in these patients. Seizure reduction was associated with the patients’ specific local CM tissue responses (individual VTAs); patients with suboptimal clinical outcomes had greater distances between the DBS electrode locations and the CM. In addition, the combination of diffusion tractography and functional connectome imaging analysis demonstrated that CM-DBS modulated a well-delineated network, mainly composed of the sensorimotor and supplementary motor cortices, brainstem, and cerebellar regions. These results suggest a modulation of the reticular system to optimally suppress seizures in patients with generalized pharmacoresistant epilepsy. Our results highlight the importance of implementing diffusion MRI in assisting the surgical targeting for DBS in pharmacoresistant epilepsy.

The CM is not only important for arousal and maintenance of consciousness but is also vital for sensorimotor coordination and the regulation of cardiac, respiratory, muscle, and reflex activities [28], making it a valuable DBS target for epilepsy treatment. Nonetheless, heterogeneity in target region and coordinates and parameters of stimulation [8, 11, 12, 29] have been used to explain the inconsistency in DBS outcomes. However, differences in DBS efficacy can be attributed to the heterogeneity in patient selection criteria and inclusion of different epileptic syndromes, further limiting results’ interpretation and agreement among the studies. Accompanying the suggestion that CM-DBS may be more effective in patients with generalized epilepsy [6] in comparison to other epilepsy syndromes, the introduction of diffusion and functional brain imaging approaches has shown to facilitate the study of neurophysiological characteristics of CM-DBS in other epileptic syndromes [7, 8]. Interestingly, to our knowledge, the current study is the first to assess both structural and functional connectivity of CM-DBS in a cohort of patients uniquely suffering from generalized pharmacoresistant epilepsy, representing a clear step forward in the understanding of CM-DBS mechanisms.

In our study, CM-DBS modulated two main fiber tracts within the reticular formation, including the brainstem-thalamo-cortical projections (ARAS) and the descending pathways to the spinal cord via the reticulospinal tracts (brainstem and superior cerebellar peduncle projections). The CM, particularly its lateral part, has reciprocal connections with the motor and primary somatosensory cortices [28]. CM also receives cholinergic and non-cholinergic (i.e., serotonergic and noradrenergic) inputs from the descending pathways. Within the descending fibers, activation of inhibitory Purkinje cells, likely results in the suppression of excitatory cerebellar output to the thalamus and thalamocortical projections, resulting in overall decreased cortical excitability[30]. Such intricated structural architecture is consistent with our structural and functional connectivity findings, supporting the key role of main efferent and afferent centromedian connections in the efficacy of CM-DBS [28], disrupting aberrant network synchrony in the reticulo-thalamo-cortical circuits [7], and interrupting or decreasing the risk of seizure activity [31], likely by inducing desynchronization and inhibition of electrical conduction through the evidenced pathways. Thus, the efficacy of DBS in epilepsy patients may be dependent on the specific cerebello-cortico-thalamic connectivity profiles of distinct thalamic subdivisions [32] and network modulation of brain states [33, 34].

The high number of projections from CM to the sensorimotor areas [28] can help explain why the representation of generalized seizures is seen in these areas in functional studies [35]. Accordingly, associations between white matter fiber connections from CM-DBS active contacts and EEG activation in the frontal, left temporal, and right anterior temporal areas and CM-DBS outcomes has been reported in patients with pharmacoresistant epilepsy [7]. Unfortunately, in the mentioned study more multifocal (*n* = 7) than generalized (*n* = 3) epilepsy patients were included, whereas significant activation during CM-DBS across the whole-cortex was detected. Further, the authors considered only fiber projections from CM to cortical sites, and electrode contacts were activated according to the seizure frequency [7]. In contrast, in our study, all patients presented a generalized seizure type, the choice of active electrode contacts, and stimulation parameters were based not only on the reduction of seizure frequency but also on the induction of delta and theta waves, characteristic of evolving rhythmic activity of seizures [12]. Moreover, our tractography approach was conducted on a whole-brain basis and combined with a discriminative fibertract analysis method [25], altogether leading to a detailed detection of fiber tracts of long-term CM-DBS outcome, involving the reticular system network. Thus, beyond patient selection, differences in DBS contact activation algorithm and whole-brain analysis could explain the different patterns of CM network connectivity and patients’ clinical responses to CM-DBS in our study.

A recent CM-DBS study, including 16 patients diagnosed with Lennox-Gastaut syndrome, used resting-state fMRI to model connectivity from electrode locations, resulting in a network composed of sensorimotor/premotor and limbic (cingulate, parahippocampal, insular) cortices, brainstem, cerebellum, and striatum (caudate, putamen) [8]. Noteworthy, the authors based their conclusions on existing indirect connections between CM and frontal cortices through the striatum [8], whereas the direct and reciprocal connections to premotor and sensorimotor cortices and to the cerebellum through the brainstem [28] were neglected, limiting the extent of their conclusions. While it is true that frontal regions are involved in the modulation of cortical processing during attention-demanding tasks [28], in Warren et al. [8], the utility of physiological recordings (e.g., intraoperative microelectrode recordings) and fMRI data is constrained by its application on anaesthetized patients. Importantly, even when we found fibers connecting to regions within the basal ganglia, including the striatum (see Supplementary file 11 Fig. 2), which act as intermediate regions connecting the CM with the prefrontal cortex [28], these fibers were not highly discriminative for the optimal outcome to DBS in our patients (see Supplementary file 11 Fig. 3). This goes well with the lack of functional connectivity to frontal areas in our analyses using the normative data. In this context, prospective studies are needed using subject-specific functional imaging to better understand this phenomena, also accounting for the possible interaction between electrode locations and functional connectivity for the optimal CM-DBS outcomes.

A previously described pattern of thalamic activation during seizures, namely, the earlier activation of CM/Pf followed by the anterior nucleus, suggests that the brainstem reticular formation could drive the generalized seizures [4]. Thus, the mechanistic effect of CM-DBS on seizure reduction in generalized epilepsy could rely on the recruitment of physiological circuits of the CM and interruption of seizure activity propagation along the cerebello-thalamo-cortical pathways. Consistently, the sensorimotor and premotor regions entrain long-range synchronization of ictal activity within the thalamocortical networks in generalized epilepsy [36]. Although the role of connectivity between the CM and brainstem/cerebellum in the antiepileptic effect of CM-DBS still needs deeper evaluation, an indirect activation of motor cortical and hippocampal regions through superomedial cerebellar cortex seems possible [37]. Therefore, DBS efficacy in pharmacoresistant patients may rely on the integrity of both the cerebellum and the superior cerebellar peduncle [38]. This, in turn, may explain the variability in the efficacy of suppressing generalized seizures when stimulating the cerebellar nuclei [38].

Despite the CM-pf complex is considered the major source of direct input to the striatum [28], our functional connectivity analysis depicted that direct connections to the brainstem/cerebellum and sensorimotor cortices were discriminative for the seizure improvement. In our patients, only few fibers were seen connecting to the subthalamic nucleus (supplementary Fig. 3); this, however, does not deny the involvement of the striatum in generalized seizures. On the contrary, it suggests that it might have a differential involvement for aberrant activity propagation and control across neurological conditions, including Lennox-Gastaut syndrome or patients with absence seizures [39], patients with several comorbid psychiatric conditions (e.g., Tourette syndrome) [40, 41], or in patients with specific gene mutations (STXBP1 and SCN2A) as evidenced in animal models of such conditions [42].

### Study Limitations

While our study provides valuable information for the detection of optimal targets for stimulation and the involved network, it does not go without limitations. First, the small sample size limits the generalizability of the study. Still, previous stimulation studies have not only used similar sample sizes but also combined epilepsy or seizure types [7, 8, 43]. The relative low incidence and prevalence of generalized pharmacoresistant epilepsy [1] and the used strict inclusion criteria make it difficult to increase the sample size in the current study, but it, indeed, turns the current study population into a unique opportunity to study the efficacy of CM-DBS. In light of this, the results may be considered preliminary, however, given the exhaustive and detailed inclusion criteria of the patients, together with the high overlap between the structural and functional connectivity, the latter coming from a normative cohort (*n* = 1000), the results are expected to have good replicability in an independent, and possibly larger, dataset.

Second, the co-registration technique of preoperative and postoperative patient images could be an additional source of methodological errors. To minimize methodological effects, also related to MRI acquisitions, we used the procedures implemented in a recently established advanced computational framework (Lead-DBS toolbox), including brain shift correction, multispectral normalization, subcortical refinement, and visual confirmation of the correct electrode placement. Finally, by using diffusion tractography, it is possible that we are reconstructing a high proportion of false-positive connections, hence, limiting the *in vivo* characterization of CM-DBS. However, the applied tractography method has been shown to achieve the greatest valid fiber connections among tractography algorithms [44]. Moreover, functional connectivity analysis, using independent normative data of 1000 young individuals, depicted a very similar network pattern. The congruency between structural and functional results strongly suggests that the identified network could play a key role in the efficacy of CM-DBS.

Since the current study only included patients diagnosed with generalized epilepsy, we cannot ensure that the modulated network is specific to generalized seizures. Nonetheless, the results evidence that the recruitment of the specific CM-driven circuits mediates the anti-seizure effect and long-term clinical outcomes of CM-DBS.

Even when no MRI evidence of anomalies affecting CM-targeting exist, it is notorious that we cannot disregard impact of structural anomalies on the connectivity profiles. However, given the evidenced correspondence between normative and individual connectivity [45] and the high overlap between our structural and functional connectivity, we can assume that the resulting networks to CM-DBS are not greatly affected by structural anomalies.

There are additional concerns regarding surgical planning and procedure. The DBS implantation procedure has not been greatly improved in the last 20 years [46]. This is besides technological advancement in brain imaging techniques, introduction of network reconstructions, and improvement in target definition with probabilistic tractography [34], none of which has yet reached the clinical routine. In our case, the implantation was guided by consensus-coordinates and electrophysiology, which has been proved highly reliable among studies [34]. Finally, while it has been largely difficult to visualize the CM nucleus or CM-Pf complex using standard MRI acquisitions, recent developments, including the use of ultra-high MRI atlasing and advanced pipelines [18, 21], currently allow for its examination in any dataset.

## Conclusion

Bilateral CM-DBS delivers significant long-term improvement in seizure frequency and quality of life in generalized pharmacoresistant epilepsy. In these patients, DBS efficacy relies on the connectivity of the CM to the brainstem and cerebellum, as well as to the sensorimotor and premotor cortices. Detailed knowledge of the disease-specific and CM-DBS-modulated networks may be an independent predictor of epilepsy patients who may benefit from DBS therapy. Further, an improved targeting within the described networks may enable the optimization of the neuromodulatory effects of CM-DBS in epilepsy patients, opening up possibilities to reduce stimulation-associated side effects or the number of non-responders. Our results evidence that a detailed study of the brain network characteristics will enhance the selection of optimal targets for stimulation among epilepsy syndromes.

## Supplementary Information

Below is the link to the electronic supplementary material.

Supplementary file1 (PDF 498 KB)

Supplementary file2 (PDF 496 KB)

Supplementary file3 (PDF 498 KB)

Supplementary file4 (PDF 497 KB)

Supplementary file5 (PDF 500 KB)

Supplementary file6 (PDF 495 KB)

Supplementary file7 (PDF 499 KB)

Supplementary file8 (PDF 497 KB)

Supplementary file9 (PDF 496 KB)

Supplementary file10 (PDF 499 KB)

Supplementary file11 (DOCX 24008 KB)

## Data Availability

Patient data, including MRI and DBS-MRI, used in this study are not publicly available due to data privacy regulations but are available for sharing with qualified investigators on reasonable request.

## Code Availability

All codes used to analyze the dataset is openly available within Lead-DBS and Lead-DBS-Connectome software (https://github.com/leaddbs/leaddbs).
