# Supplementary material for: Network Substrates of Centromedian Nucleus Deep Brain Stimulation in Generalized Pharmacoresistant Epilepsy
Source: Neurotherapeutics. 2021 Apr 26;18(3):1665–77. doi: 10.1007/s13311-021-01057-y (PMC8608991; doi:10.1007/s13311-021-01057-y)
Supplement: Supplementary file 1 — Supplementary file1 (PDF 498 KB) [file 13311_2021_1057_MOESM1_ESM.pdf]

Please wait...

If this message is not eventually replaced by the proper contents of the document, your PDF viewer may not be able to display this type of document.

You can upgrade to the latest version of Adobe Reader for Windows®, Mac, or Linux® by visiting http://www.adobe.com/products/acrobat/readstep2.html.

For more assistance with Adobe Reader visit http://www.adobe.com/support/products/acrreader.html.

Windows is either a registered trademark or a trademark of Microsoft Corporation in the United States and/or other countries. Mac is a trademark of Apple Inc., registered in the United States and other countries. Linux is the registered trademark of Linus Torvalds in the U.S. and other countries.

Springer
http://www.springer.com/journal/13311

Neurotherapeutics
The Journal of the American Society for Experimental Neurotherapeutics
Editor-in-Chief: Mouradian, M.
ISSN: 1933-7213 (print version)
ISSN: 1878-7479 (electronic version)
Journal no. 13311

http://www.springer.com/journal/13311

Neurotherapeutics
The Journal of the American Society for Experimental Neurotherapeutics
Editor-in-Chief: Mouradian, M.
ISSN: 1933-7213 (print version)
ISSN: 1878-7479 (electronic version)
Journal no. 13311
