# Supplementary material for: Network Substrates of Centromedian Nucleus Deep Brain Stimulation in Generalized Pharmacoresistant Epilepsy
Source: Neurotherapeutics. 2021 Apr 26;18(3):1665–77. doi: 10.1007/s13311-021-01057-y (PMC8608991; doi:10.1007/s13311-021-01057-y)
Supplement: Supplementary file 11 — Supplementary file11 (DOCX 24008 KB) [file 13311_2021_1057_MOESM11_ESM.docx]

**Network substrates of centromedian nucleus deep brain stimulation in generalized pharmacoresistant epilepsy**

**Running head:** CM-DBS outcome networks in generalized epilepsy

Cristina V. Torres Diaz^1*†^, Gabriel González-Escamilla^2*†^, Dumitru Ciolac^2,3,4^, Marta Navas García^1^, Paloma Pulido Rivas^1^, Rafael G. Sola^1^, Antonio Barbosa^5^, Jesús Pastor^6^, Lorena Vega-Zelaya^6^, Sergiu Groppa^2^

^1^Department of Neurosurgery, University Hospital La Princesa, Madrid, Spain.

^2^Movement Disorders and Neurostimulation, Department of Neurology, Focus Program Translational Neuroscience (FTN), Rhine Main Neuroscience Network (rmn2), University Medical Center of the Johannes Gutenberg University Mainz, Mainz, Germany

^3^Laboratory of Neurobiology and Medical Genetics, Nicolae Testemitanu State University of Medicine and Pharmacy, Chisinau, Republic of Moldova

^4^Department of Neurology, Institute of Emergency Medicine, Chisinau, Republic of Moldova

^5^Department of Neuroradiology University Hospital La Princesa, Madrid, Spain.

^6^Department of Clinical Neurophysiology University Hospital La Princesa, Madrid, Spain.

^†^Authors contributed equally to this work.

**Supplementary information**


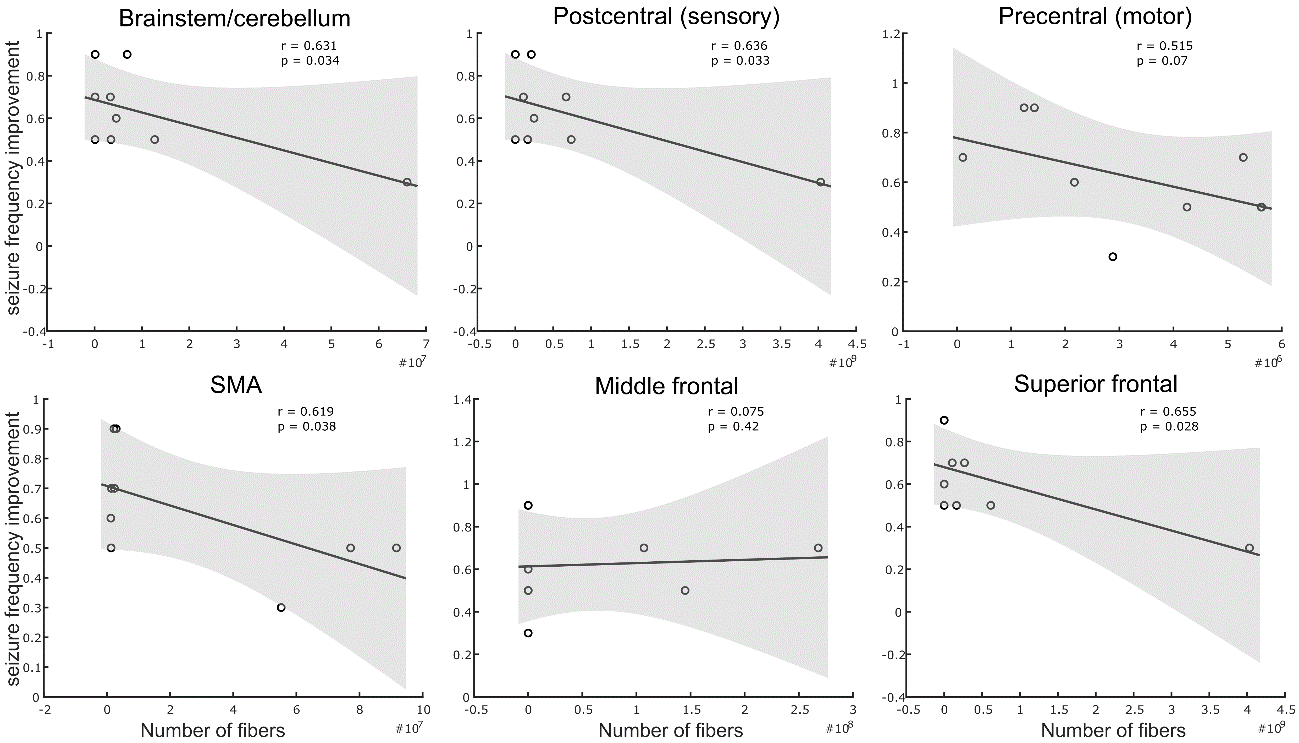


**Supplementary Figure 1**. Fiber association to CM-DBS outcome after excluding non-responsive patient. Regression plots for the association between centromedian nucleus (CM) deep brain stimulation (DBS) and seizure frequency improvement with (**A**) brainstem, (**B**) postcentral (sensorial) cortex (**C**) precentral (motor) cortex, (**D**) supplementary motor area (SMA), (**E**) middle frontal gyrus, and (**F**) superior frontal cortex. All associations were conducted using independent general lineal models. Blue shaded areas represent 95% confidence intervals. Notice that the effects have remains for all regions in comparison to the analyses including all 10 patients, except for the lack of associations in the middle frontal gyrus.


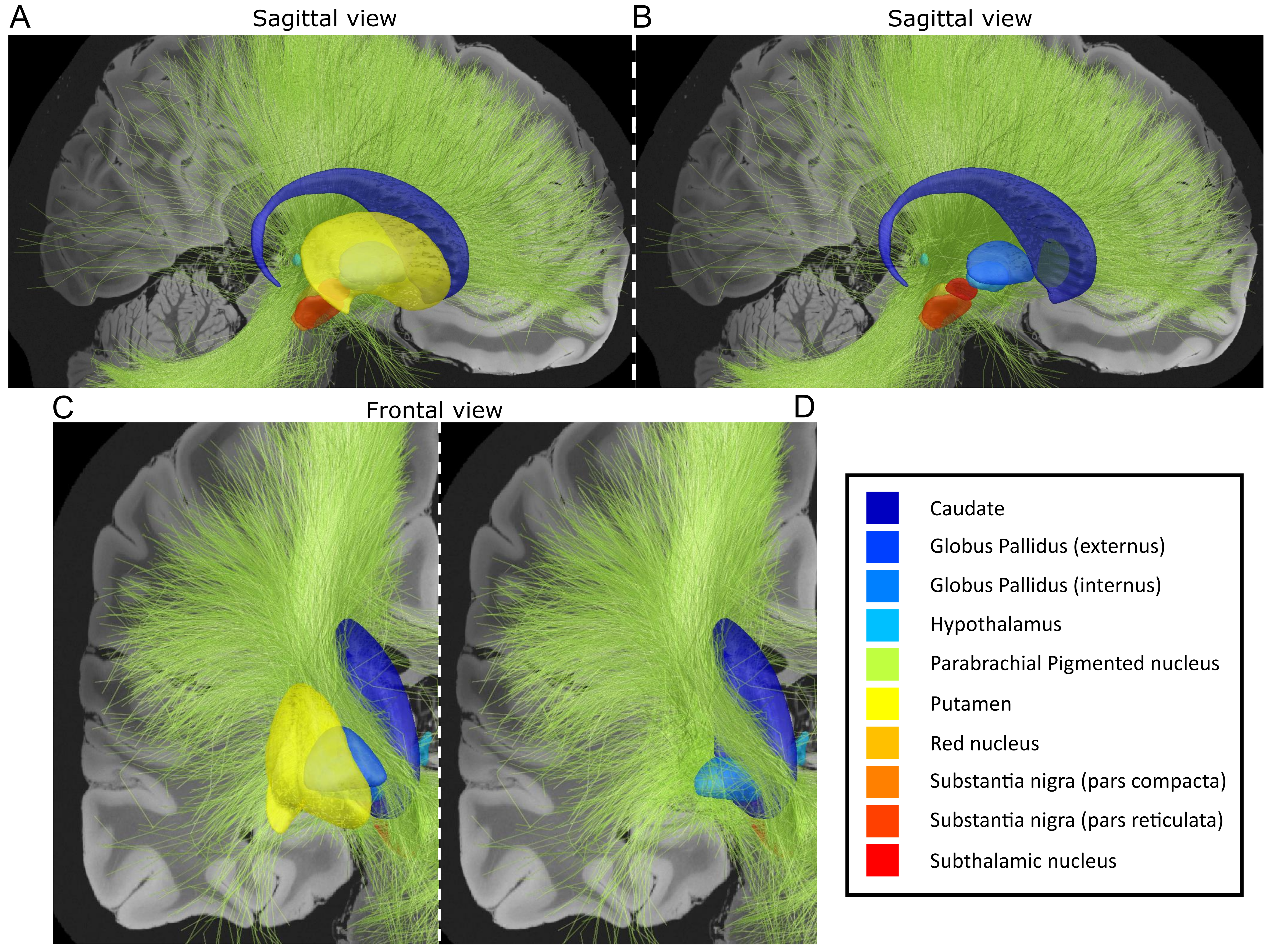


**Supplementary Figure 2**. All fibers connected to the volumes of tissue activated (VTAs) across patients depicting regions of the basal ganglia (BG) as defined in the CIT168 atlas (Pauli et. al., 2017). **A)** Sagittal view of the connected fibers showing the basal ganglia nuclei. **B)** Depicts the same sagittal view as in **A** without showing the putamen and globus pallidus externus to allow visibility of the deeper nuclei. **C)** Coronal view of the connected fibers displaying all basal ganglia corresponding to **A**. **D)** Depicts the same coronal view as in **C** without showing the putamen and globus pallidus externus to allow visibility of the deeper nuclei. In brief, all BG nuclei except for the globus pallidus externus were crossed by connected fibers. From all structures the globus pallidus internus, subthalamic nucleus, and caudate have the most observable fibers, whereas the fibers ascending to the frontal cortex cross laterally the putamen.


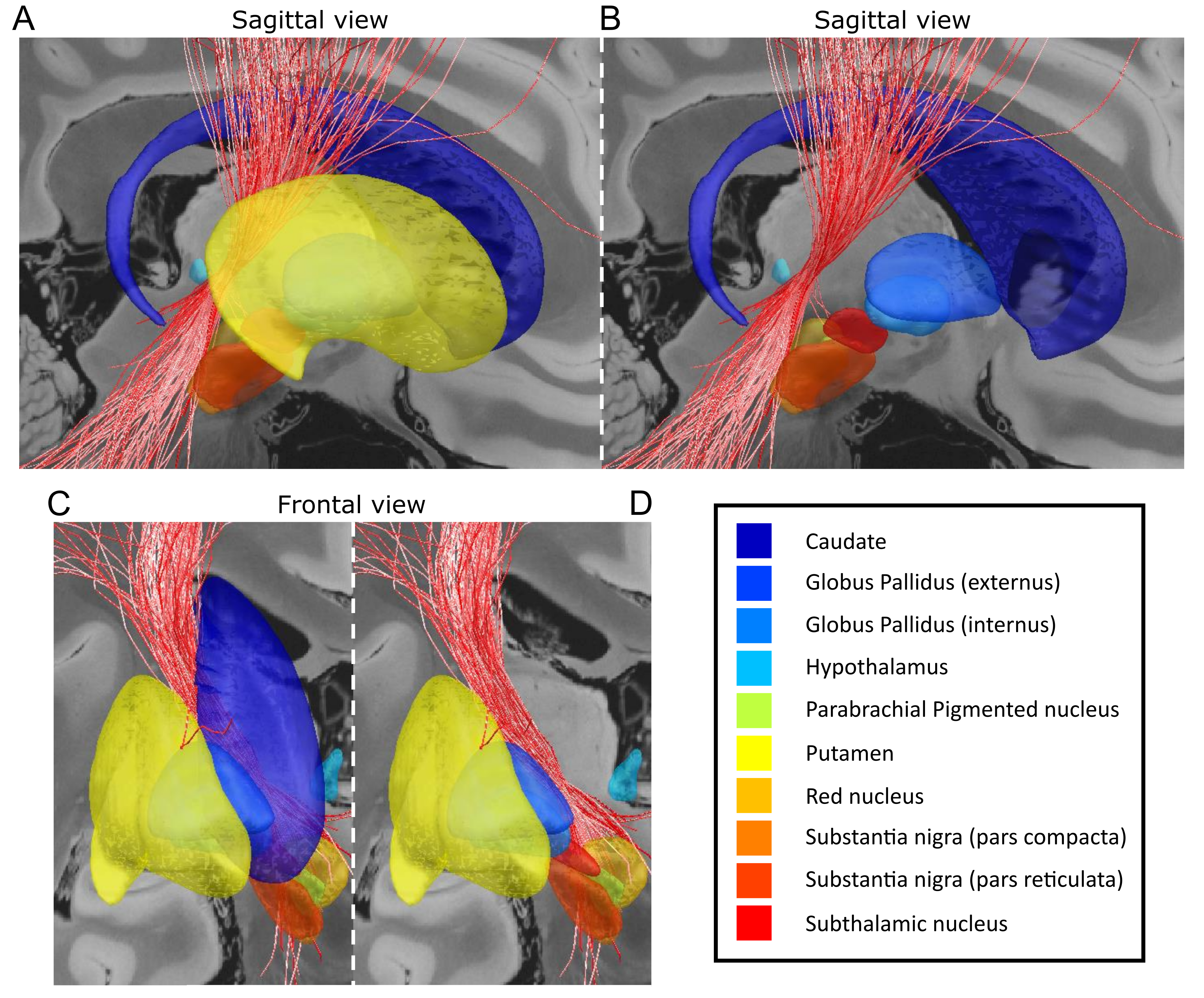


**Supplementary Figure 3**. Discriminative fibers associated with clinical seizure improvement depicting all regions of the basal ganglia (BG) as defined in the CIT168 atlas (Pauli et. al., 2017). **A)** Sagittal view of the discriminative fibers showing the basal ganglia nuclei. **B)** Depicts the same sagittal view as in **A** without showing the putamen to allow visibility of the deeper nuclei. **C)** Coronal view of the discriminative fibers displaying all basal ganglia corresponding to **A**. **D)** Depicts the same coronal view as in **C** without showing the caudate and globus pallidus externus to allow visibility of the deeper nuclei. In brief, it is observable that all discriminative fibers connected the CM directly to the sensorimotor cortices without crossing the BG. In **A** and **B** it is observable that the fibers go posterior to the BG, while in **C** and **D** its visible that fibers go medially to BG nuclei. The exception is that few fibers are seen to connect with the subthalamic nucleus (seen in **B**).

**Supplementary references**

Pauli WM, Nili AN, Tyszka JM. A high-resolution probabilistic in vivo atlas of human subcortical brain nuclei. Sci Data 2018;5:180063.
